# The role of psycholinguistics for language learning in teaching based on formulaic sequence use and oral fluency

**DOI:** 10.3389/fpsyg.2022.1012225

**Published:** 2022-09-09

**Authors:** Yue Yu

**Affiliations:** School of Foreign Studies, Nanjing University, Nanjing, China

**Keywords:** formulaic sequence, oral fluency, speed fluency, breakdown fluency, repair fluency, psycholinguistics, proceduralization

## Abstract

Psycholinguistics has provided numerous theories that explain how a person acquires a language, produces and perceives both spoken and written language, including theories of proceduralization. Learners of English as a foreign language (hereafter referred to as EFL learners) often find it difficult to achieve oral fluency, a key construct closely related to the mental state or even mental health of learners. According to previous research, this problem could be addressed by the mastery of formulaic sequences, since the employment of formulaic sequences could often promote oral fluency in the long run, reflected in the positive relationship between formulaic sequence use and oral fluency. However, there are also findings contradicting the abovementioned ones, without adequate explanations. This study aims to explore the roles of formulaic sequences in oral fluency, taking into account the relationship between formulaic sequence use and oral fluency. This study investigated 120 pieces of spoken narratives by Chinese EFL learners, using both quantitative and qualitative methods, combined with artificial intelligence techniques. Results of canonical correlation analysis showed that the frequency of formulaic sequences was significantly related to speed fluency (*r* = 0.563, *p* = 0.000) and breakdown fluency (*r* = 0.360, *p* = 0.001), while the variety of formulaic sequences was significantly related to repair fluency (*r* = 0.292, *p* = 0.035). Case studies further demonstrated that formulaic sequences could contribute to oral fluency development by promoting speed and reducing pausing when retrieved holistically, but they sometimes lost processing advantages when retrieved and processed in a word-by-word manner. The inappropriate use of formulaic sequences also neutralized the facilitative effects of formulaic sequences on repair fluency and could mirror speakers’ occasional tendency to sacrifice repair fluency for the improvement of speed and breakdown fluency when using formulaic sequences. Pedagogical implications were provided accordingly to promote sustainable oral fluency development through the use of formulaic sequences.

## Introduction

Traditionally speaking, in language learning and teaching, teachers and students have been pursuing three goals, namely fluency, nativeness, and intelligibility (Suzuki and Kormos, 2020). In this sense, a second language (hereafter referred to as L2) learner or learner of English as a foreign language (hereafter referred to as EFL learner and used interchangeably with L2 learner) could never be regarded as mastering the language without becoming a fluent speaker. Moreover, failure to maintain fluency would lose the listeners’ attention as well as the speakers’ face (Lennon, 2000). In this sense, oral fluency could be related to speakers’ mental state, or even mental health, further emphasizing the crucial role of fluency in communication and interaction. Many second language learners actually consider L2 fluency as “the ultimate personal dream,” the key to the ability of effective communication (Tavakoli and Wright, 2020, p. 1). All in all, fluency is crucial to language learners, and is widely researched in recent years (Song and Li, 2020; Feng and Guo, 2022).

However, the pursuit of fluency is never a piece of cake. Instead, it could be a hot potato both for teachers and students. Although many people may have certain knowledge of another language which is not their mother tongue, they are usually much less fluent in their second language than their first language, and there is a “fluency gap” (Segalowitz, 2010, p. 2). People have been frustrated about this fluency gap, sparing no effort to study it, trying their best to know how to bridge this gap.

Some scholars have claimed that a possible solution to the problem of oral fluency is the mastery of formulaic language, retrieved from long-term memory as if they were single words (Nattinger and DeCarrico, 1992; Towell et al., 1996; Chambers, 1998; Wray, 2002). The idea that formulaic language can help language users become more fluent was first outlined by Pawley and Syder (1983), who claimed that the storage of formulaic language in long-term memory can make up for limited working memory. Some scholars even regard mastery of formulaic language as a prerequisite for learners to attain a native-like command of the language, because of their functions to produce natural, idiomatic, and native-like stretches of discourse, and facilitate fluency (Pawley and Syder, 1983; Dechert, 1984; Skehan, 1998; Wray, 2002; Granger and Paquot, 2008). In fact, formulaic language may serve as a “shortcutting device,” which saves time and effort of processing, and enables the speaker to pay attention to other things besides language processing (Peters, 1983, p. 3). All these reflect the roles of formulaic sequences in language learning through the mechanism of proceduralization. Many studies have indeed showcased the importance of formulaic sequences in language use (Boers and Webb, 2018; Wang, 2018; Liu and Chen, 2020; Shin, 2020; Vercellotti et al., 2021; Fritz et al., 2022; Kim and Kessler, 2022).

The findings of previous research did demonstrate the significant influence of formulaic language on the natural and fluent proficiency of English (Russell, 2017), or specifically oral fluency. On the other hand, results of previous research on the relationship between the use of formulaic sequences and oral fluency were not always consistent. Some studies found that the correlations between indices of formulaic sequences and oral fluency did not always reach a significant level (e.g., Yan, 2020). However, none of them elaborated on the reasons for the contradicting phenomena, or explored when, where, why, and how could formulaic sequences promote different dimensions of fluency so as to help maintain the sustainable oral fluency development in different aspects.

To fill these research gaps, the present study was carried out to explore the roles of formulaic sequences in the three dimensions of oral fluency, namely speed, breakdown, and repair fluency, reflected in Chinese EFL learners’ spoken narratives. The positivity of the proposed work consists in the fact that the present study investigated sophomore English majors in China, whose treatment of instructions was similar to each other, so that the proposed work could better showcase the influences of formulaic sequences on oral fluency under similar circumstances. Results of the present study systematically displayed the correlations between indices of formulaic sequences and the three dimensions of oral fluency, and illustrated the conditions on which formulaic sequences could contribute to oral fluency development by promoting speed and reducing pausing, as well as the situations on which they lost processing advantages. Based on the relationship between formulaic sequence use and oral fluency, the present study could help demonstrate the role of psycholinguistic theories related to proceduralization, in language learning. Such findings also helped students improve their oral fluency by learning formulaic sequences.

## Literature review

### Formulaic sequence

In previous research, the term “formulaic sequence” was often used interchangeably with “formulaic language,” as an umbrella term (Weinert, 2010; Wood, 2015; Lu and Deng, 2019), which received opposition in recent years, especially in the context of SLA. Jeong and Jiang (2019) defined formulaic sequence in a specific sense, as a multiword sequence that forms a complete phrase, which should be distinguished from a lexical bundle, which runs across phrasal boundaries. Their definition of formulaic sequences echoes Qi and Ding’s (2011) version, which considers formulaic sequences to have “syntactically and semantically well-formed structure,” “with a complete syntactic structure and semantic meaning that can be found in an authoritative dictionary” (p. 165). In addition, Jeong and Jiang’s (2019) research reported the processing advantage of formulaic sequences, but the results showed no processing advantage of lexical bundles (Jeong and Jiang, 2019). In other words, only formulaic sequences, which do not run across phrasal boundaries, were reported to be processed faster.

The most widely employed definition of formulaic sequence is by Wray (2002), who defined a formulaic sequence as follows:

a sequence, continuous or discontinuous, of words or other elements, which is, or appears to be, prefabricated: that is, stored and retrieved whole from memory at the time of use, rather than being subject to generation or analysis by the language grammar. (p. 9)

However, this definition was later regarded as a stipulative and not operational one (Wray, 2009), and that the holistic storage and representation are controversial and contradicting. Meanwhile, this definition reflects the fuzzy nature of formulaic sequences, since a word sequence processed holistically by one person may not be processed holistically by another, and the matter of holistic processing may be a matter of degree (Boers et al., 2006).

In her later work, Wray (2008) further defined formulaic sequences as words that have “an especially strong relationship with each other in creating their meaning” (p. 9), and distinguished between speaker-external and speaker-internal approaches to formulaicity, the former of which focused on the formal properties of strings, frequency of occurrence, or pragmatic functions of formulaic sequences, while the latter of which focused on the holistic retrieving and storage of formulaic sequences. In this sense, two prominent features of formulaic sequences, namely their ability to be holistically stored and retrieved, as well as the connections between the words constituting a formulaic sequence, actually correspond to the speaker-internal and speaker-external perspectives, respectively.

Based on this distinction, Myles and Cordier (2017) emphasized the necessity to re-term these two approaches to formulaic sequences, regarding them as two distinct constructs which are conceptually fundamentally different, indicating an external linguistic phenomenon and an internal cognitive process, respectively. On one hand, they re-termed speaker-external formulaic sequences as linguistic clusters (LC), defined as “multimorphemic clusters which are either semantically or syntactically irregular, or whose frequent co-occurrence gives them a privileged status in a given language as a conventional way of expressing something” (Myles and Cordier, 2017, p. 12). On the other hand, they re-termed speaker-internal formulaic sequences as processing units (PU), defined as “a multiword semantic/functional unit that presents a processing advantage for a given speaker, either because it is stored whole in their lexicon or because it is highly automatized” (Myles and Cordier, 2017, p. 12).

The present study adopts the speaker-external approach. The main reason is that the speaker-external approach generally involves the phraseological approach regarding the identification of formulaic sequences, which is essentially meaningful, since apart from frequency there should be other features that make formulaic language formulaic (Xuan et al., 2021). The speaker-external approach can be of more use to language learners and teachers, since this could ensure the close association between the constituent words of the selected sequences, and these sequences would be relatively easier to teach, learn, or memorize. As the present study mainly focuses on the speaker-external approach, all the formulaic sequences in the present study are linguistic clusters (LC), but they do not necessarily function as processing units (PU). Only those proceduralized phrases could function as both processing units (PUs) and linguistic clusters (LCs), while those non-proceduralized phrases function only as linguistic clusters (LCs), but not processing units (PUs).

The present study combines the definition by Wray (2002, 2008), Jeong and Jiang (2019) and Qi and Ding (2011), focusing more on the speaker-external approach, treating formulaic sequences as linguistic clusters, and defines formulaic sequences as a sequence of words, continuous or discontinuous, with a syntactically and semantically well-formed structure, which has a semantically clear meaning, does not run across phrasal boundaries, and whose usage can be found in an authoritative dictionary.

### Oral fluency

Tavakoli and Hunter (2018) raised a model of fluency, which contained four different but interrelated levels, namely very broad, broad, narrow and very narrow. In this model, fluency is regarded as a pyramid. From the bottom to the top, the four levels indicate all kinds of L2 ability, L2 speaking ability, the flow and continuity of speech, and concrete and measurable features of fluency. The present study only focuses on the very narrow sense of fluency in Tavakoli and Hunter’s (2018) model, investigating the temporal aspects, which also belongs to the narrow sense of fluency in Lennon’s (1990, 2000) model. The reason is that the measurement of the broad sense of fluency in Lennon’s (1990, 2000) model, as well as of the very broad sense and broad sense of fluency in Tavakoli and Hunter’s (2018) model usually relies on the perceptions of L1 listeners, or some overall speaking tests, which may not tease out fluency from other L2 speaking skills. The narrow sense of fluency in Tavakoli and Hunter’s (2018) framework concentrates on L2 oral fluency, but may not be put into systematic and objective measurement as convenient as the very narrow sense of fluency.

Different from the broad-narrow division, Segalowitz’s (2010) triadic model explores the different sense of fluency from another approach, dividing fluency into three senses, namely cognitive fluency, utterance fluency, and perceived fluency. Cognitive fluency concerns the “ability to efficiently mobilize and integrate the underlying cognitive processes responsible for producing utterances with the characteristics that they have”; utterance fluency indicates the “features of an utterance,” which are the “actual properties of the utterance,” and are “the fluency characteristics that a speech sample can possess”; perceived fluency is “a judgment made about speakers based on impressions drawn from their speech samples” (Segalowitz, 2010, p. 48). These three senses of fluency are internally related, with studies suggesting the strong link between utterance fluency and cognitive fluency (e.g., Kahng, 2014), as well as between utterance fluency and perceived fluency (e.g., Bosker et al., 2014) for both native and nonnative speakers.

The present study only focuses on utterance fluency. The reason is that only this sense of oral fluency has a concrete and measurable nature, allowing objective and systematic examination and measurement based on the acoustic characteristics of speech (Duran-Karaoz and Tavakoli, 2020; Tavakoli and Uchihara, 2020), and it is especially “amenable to quantitative methods” as well as enables “a degree of standardization and comparison across studies” (Tavakoli et al., 2020, p. 170).

Utterance fluency, which is the focus of the present study, can be further divided into two aspects, namely speed and smoothness. Related to these two aspects of fluency is another triadic framework of fluency constructed by Skehan (2003) as well as Tavakoli and Skehan (2005). This model divides fluency into three aspects, including: (a) speed fluency, which is the flow and continuity of speech, as well as how fast a speaker could talk; (b) breakdown fluency, which involves the pauses that disrupt the flow of speech; and (c) repair fluency, which concerns how much a speaker corrects, reformulates, and restores utterances. Later, Skehan (2014) drew a distinction between the speed of speech (speech rate) and that of the disturbance of the flow of speech (pausing and reformulations). The former is associated with speed fluency, correspondent with the aspect of speed, while the latter is associated with breakdown fluency and repair fluency, correspondent with the aspect of smoothness mentioned in the previous paragraph. Measures of speed, breakdown, and repair also constitute the very narrow sense of fluency in the abovementioned Tavakoli and Hunter’s (2018) model.

The present study adopts the aforementioned triadic framework of fluency constructed by Skehan (2003) as well as Tavakoli and Skehan (2005), and focuses on speed, breakdown, and repair fluency, exploring how the employment of formulaic sequences could help sustain the development of these three dimensions of oral fluency.

### The relationship between formulaic sequence use and oral fluency

#### Theoretical basis

**Figure 1:**
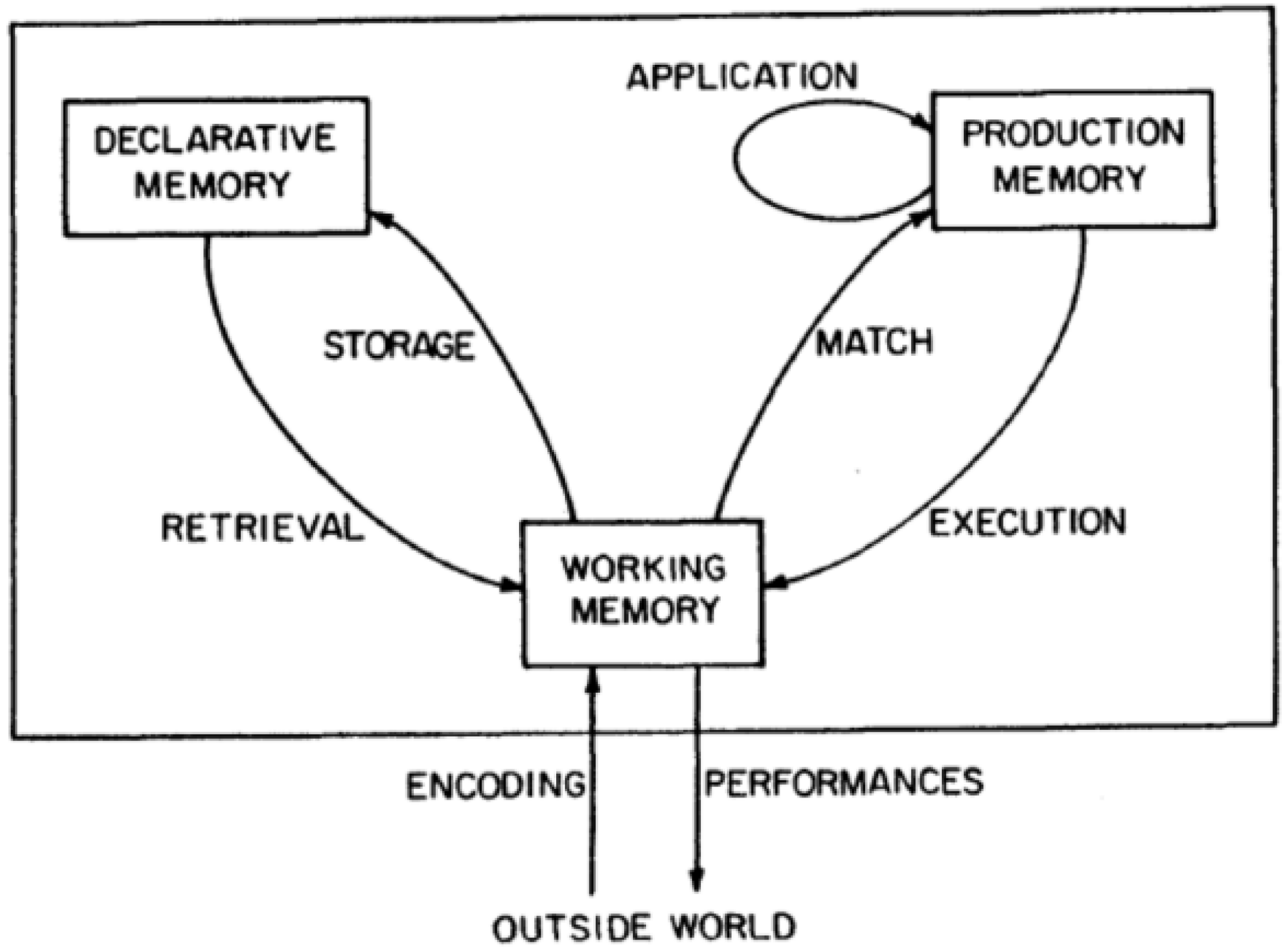
Framework for the ACT (Anderson, 1983, p. 19).

The relationship between the use of formulaic sequences and oral fluency has psycholinguistic basis. The facilitative effect of formulaic sequences on oral fluency could be illustrated from a cognitive perspective, concentrating on proceduralization. Anderson’s (1983) ACT model emphasizes the importance of proceduralization (see Figure 1 for details). This model regards working memory as crucial to language development. However, working memory has a very limited capacity, while declarative knowledge requires a lot of attention and takes up much more space than procedural knowledge, which does not require focal attention, as procedural knowledge could be processed by working memory in larger units, not surpassing the capacity of working memory. Viewed in this light, speech production, which requires rapid performance, calls for conversion of declarative knowledge into procedural knowledge. The transformation of declarative knowledge into procedural knowledge is called proceduralization, including three stages, namely cognitive, associate, and autonomous stages. First, in the cognitive stage, learners are involved in conscious activities, acquiring typical declarative knowledge that can usually be described verbally. Second, in the associate stage, learners detect and correct the errors and mistakes in the original declarative knowledge, which starts to be proceduralized. They also gradually convert condition-actions pairs, originally in declarative forms, into production sets, while keeping the initial declarative representation. Third, in the autonomous stage, learners rely less on working memory, but perform more subconsciously and automatically, and make fewer errors and mistakes. This automatization signals the completion of proceduralization.

The holistic hypothesis could reflect the issue of proceduralization, which holds that formulaic sequences are stored as single units that are directly addressable and retrievable from the mental lexicon and that they have been lexicalized (Peters, 1983; Weinert, 1995; Wray, 2002). Pawley and Syder (1983) believed that as single memorized units, formulaic sequences are processed more quickly and easily than the sequences of words generated creatively. The mind uses the abundant resources of long-term memory to store prefabricated chunks that can be directly used in language production. This compensates for the limited resources of working memory, preventing it from being overloaded. In this way, the use of formulaic sequences can reduce the load on the attentional resources, or in other words, free up the attentional resources for other aspects of language production, reduce the amount of language planning and processing, thereby facilitating oral production (Kormos, 2006; Skehan, 2009, 2014; Siyanova-Chanturia and Van Lancker Sidtis, 2018; Tavakoli and Uchihara, 2020). In other words, formulaic sequences have a processing advantage, indicating that they are processed faster than control sequences (Jiang and Nekrasova, 2007; Conklin and Schmitt, 2008; Siyanova-Chanturia et al., 2011; Tremblay et al., 2011).

The issue of proceduralization is also reflected in the language production model. According to the language production model (Levelt, 1989), language production includes three stages, namely conceptualization, formulation, and articulation. The use of formulaic sequences could facilitate speech production in the formulation and articulation stages. In the formulation stage, grammatical encoding, which usually involves automatic processing, could be further promoted by the use of formulaic sequences. Since formulaic sequences are usually proceduralized, speakers with a larger repertoire of formulaic sequences can retrieve longer units of phrases, helping them “buy” processing time for the preparation of other processing needs (Skehan, 1998; Boers et al., 2006). In addition, these speakers are less likely to show hesitations when using the formulaic sequences because of the relatively fixed structure, which makes them less likely to be greatly modified in usage (Boers et al., 2006). Then, in the articulation stage, formulaic sequences are usually pronounced faster, in an automatized way (Kormos, 2006; Skehan, 2014).

#### Empirical studies

Most of the previous studies investigated the relationship between the use of formulaic sequences and oral fluency from a quantitative approach. They usually designed experiments to investigate participants’ processing of formulaic sequences, or calculated the correlations between indices of formulaic sequences and those of oral fluency. Quantitative analysis in previous research employed different measurement of oral fluency when investigating the relationship between the use of formulaic sequences and oral fluency.

Some of the previous studies employed subjective judgment to measure fluency, so what they actually examined was the relationship between the use of formulaic language and perceived fluency. Boers et al. (2006) investigated 32 English majors’ performance in a speaking task, and the results showed a significant positive relationship between the number of formulaic sequences and their oral fluency scores. Stengers et al. (2011) investigated the retell task performance of 60 L1 Dutch learners, 26 of whom were L2 English learners and 34 of whom were L2 Spanish learners, and they found a significant positive relationship between the number of formulaic sequences and the oral fluency scores, leading to their conclusion of the processing advantage of mastery of formulaic sequences, not only for native speakers but also for L2 learners, especially for L2 English learners compared with L2 Spanish learners. Saito (2020) investigated the degree to which 85 Japanese EFL learners’ collocation use was related to first language raters’ intuitive judgments of second language speech in a picture description task. The results showed that the natural use of more infrequent multi-word units has a strong impact on perceived L2 oral fluency in a broad sense.

As for utterance fluency in contrast to perceived fluency, some other studies employed objective measurement to measure the speed of speech, focusing on the relationship between the use of formulaic language and speed fluency. Wood (2009, 2010) found that in long-term learning process, the improvement of oral fluency measured by several indices (like speech rate, articulation rate, phonation time ratio, and mean length of run) was usually accompanied by the increase of formulaic sequences. Thomson (2017) found that the use of multiword expressions was positively correlated with speech rate in L2 English dialogues of 73 students in Japan, reaching significant levels in pretest, but not in posttest. McGuire and Larson-Hall (2017) investigated 19 L2 English speakers’ use of formulaic sequences and oral fluency, and they found a moderate or medium relationship between the use of formulaic speech and oral fluency. Xuan et al. (2021) investigated the use of English formulaic language in the oral performance of 86 L1 Chinese L2 English learners, and they found a significant relationship between the use of formulaic language and oral English fluency. Also, the use of two-word formulaic sequences contributed most to this relationship compared with the use of three-word lexical bundles and four-word lexical bundles.

There are also several studies that involved breakdown fluency (pausing) when exploring the relationship between the use of formulaic language and oral fluency. Tavakoli (2011) compared the performance of L2 English learners and native English speakers in a picture description task, and the results showed that L2 English learners paused more frequently than native English speakers, and the use of formulaic sequences could reduce the pauses and thus facilitated oral fluency. Yan (2020) investigated the relationship between the use of formulaic sequences and oral fluency on elicited imitation tasks, and the results showed that formulaic sequences significantly reduced the number of pauses, but did not influence the speech rate.

Compared with speed fluency and breakdown fluency, repair fluency was not often involved regarding the relationship between the use of formulaic language and oral fluency. Only a few studies have taken repair fluency into consideration. Tavakoli and Uchihara (2020) investigated 56 learners’ performance in a spoken narrative task, and their results showed the positive correlation between high-frequency n-grams and articulation rate, the negative correlation between n-gram proportion and the frequency of mid-clause pauses, the positive correlation between n-gram association strength and the frequency of end-clause pauses, as well as the negative correlation between n-gram association strength and repairs. The so-called n-grams in their studies encompassed formulaic sequences. Ogawa’s (2021) study found that formulaic language instructions significantly increased students’ speed fluency measure (mean length of run), but had no significant impact on breakdown or repair fluency measures.

As such, most of the previous research has reported a positive correlation between the use of formulaic language and overall oral fluency. Since formulaic sequences fall into the scope of formulaic language, to a large degree, the positive relationship between the use of formulaic language and oral fluency could mirror the positive relationship between the use of formulaic sequences and oral fluency. These results could correspond well to the findings that formulaic sequences play a crucial role in language use (Boers and Webb, 2018; Wang, 2018; Liu and Chen, 2020; Shin, 2020; Vercellotti et al., 2021; Fritz et al., 2022; Kim and Kessler, 2022).

On the other hand, results of previous research on the relationship between the use of formulaic sequences and oral fluency were not always consistent. Some studies found that the correlations between indices of formulaic sequences and oral fluency did not always reach a significant level. For instance, Yan (2020) reported no significant relationship between the use of formulaic sequences and speech rate. However, partly due to the lack of qualitative approach, none of the studies explained the reasons for these conflicting phenomena. Qualitative approach has been proved to be helpful in research on fluency (Barzegar and Fazilatfar, 2019), as well as in the analysis of speakers’ use of words in speech (Allami and Barzegar, 2020). There has been a calling for more qualitative studies concerning formulaic sequence use or oral fluency (Nergis, 2021). Thus, qualitative analysis was supplemented in this study to further investigate the relationship between formulaic sequence use and oral fluency, helping to solve the confliction of previous findings.

Also, previous research hardly explored when, where, why, and how could formulaic sequences promote different dimensions of fluency so as to help maintain sustainable oral fluency development in different aspects. Traditional models of the relationship between formulaic sequence use and oral fluency usually treated oral fluency as a whole, or only focused on some of the aspects of oral fluency. However, speed, breakdown, and repair fluency are the three key components indispensable to oral fluency, and their different features suggested the possibility that they would make different contributions to the relationship between formulaic sequence use and oral fluency, thereby deserving more research (Huensch and Tracy-Ventura, 2017; Magne et al., 2019). Thus, the proposed model in the present study would take all three dimensions of oral fluency into consideration, so as to provide richer and clearer findings about the relationship between formulaic sequence use and oral fluency.

## Research questions

This study aims to investigate the relationship between formulaic sequence use and oral fluency from both quantitative and qualitative perspectives. Specific research questions were:

1. To what extent is formulaic sequence use related to speed fluency?
2. To what extent is formulaic sequence use related to breakdown fluency?
3. To what extent is formulaic sequence use related to repair fluency?

## Methodology

### Data source

The Test for English Majors-Band 4 (hereafter referred to as TEM-4; oral) is a standardized proficiency test held in China to measure oral proficiency of English majors in the sophomore year. The TEM-4 oral test includes three tasks, often on the similar or related topics. The second task, used to elicit data in the present study, is a spoken narrative task in which the students are required to talk on a given topic. Students are required to prepare for 3 min and then talk for 3 min. This preparation time is regarded as an opportunity to “reduce the cognitive load and communicative pressure of the task” by providing students with an opportunity to make a plan about the content of their talk (Tavakoli and Uchihara, 2020, p. 513). The present study chose the second task as it was the only task that could reflect test-takers’ own productions of formulaic sequences, different from the retelling task (Task 1) and dialogic discussion task (Task 3).

Test-takers are rated on a 100-point scale based on five sub-scales. The overall numeric score is eventually transformed into one of the four ranks considering their positions among all speakers based on numeric scores. The highest is Rank 4, featuring “excellent” speakers. The second is Rank 3, featuring “good” speakers. The third is Rank 2, featuring “qualified” speakers. The last is Rank 1, featuring “unqualified” speakers.

The present study selected recordings from 2021’s TEM-4 as the data. The present study focused on Task 2, and the topic of Task 2 of 2021’s TEM-4 oral test was to “recall an experience in which you made a mistake and eventually put it right.” Test-takers were required to prepare for 3 min and talk for 3 min as has been mentioned.

The present study sampled 120 recordings of the TEM-4 oral test in 2021, together with a detailed list of scores, as well as three separate recordings of performance in three tasks by all the students taking TEM-4 oral test in 2021. Names and other private/confidential information of the test-takers were not shown. While these students’ overall scores and ranks were based on the assessment of their performance in all three tasks, the data used in the present study came from their performance of only Task 2 (i.e., a spoken narrative task). The data set contained 120 task performance, 30 from each Rank, totaling 362 min of recordings [i.e., 120 × (3 min ± 5 s)].

### Data-analysis

#### Measuring formulaic sequences

Before the measurement of formulaic sequences, all the recordings were automatically transcribed with the assistance of artificial intelligence techniques, using the online transcription tool *Otter*,^1^ which is a useful tool to provide automatic transcriptions used in previous research (Chang and Windeatt, 2021). I also manually edited the transcripts to revise incorrectly transcribed words, so as to ensure the accuracy of transcription. In the present study, the 120 transcripts contain 38,288 words in total. Meaningless words like “uh, um” were not transcribed, and were not counted into the total number of words mentioned above.

The identification of formulaic sequences was completed manually according to a set of standards, including:

1. An FS should be composed of two or more than two words;
2. An FS should be at the phrasal or clausal level;
3. An FS should be contained in the Longman Dictionary of Contemporary English.^2^

All the formulaic sequences were coded with BFSU Qualitative Coder. The whole data set was coded separately by three coders. A major issue with the phraseological approach to measuring formulaic sequences is its involvement of “a fair degree of subjectivity” (Tavakoli and Uchihara, 2020, p. 516), resulting in the low agreement between trained judges (*r* < 0.60 in Boers et al., 2006 and Stengers et al., 2011), largely lower than the median interrater reliability in SLA research (*r* = 0.92 in Plonsky and Derrick, 2016). Thus, after the coding process of the present study was completed by three coders, we did not calculate a coefficient rate and then move on. Instead, all the discrepancies were discussed until the agreement was reached.

Then, BFSU Qualitative Explorer was used to calculate the number of formulaic sequences. The frequency, proportion, and variety of each type of formulaic sequences were calculated, since these three indices were shown to be related to oral fluency. Frequency refers to the total output of formulaic sequences in the given time, or in other words, the total number of formulaic sequences in each text transcribed from every test-taker’s recording, reflecting the overall quantity of formulaic sequences employed by speakers; proportion refers to the ratio of formulaic sequences produced in each text, which is the total number of formulaic sequences divided by the total number of words in each text, reflecting speakers’ degree of tendency to use formulaic sequences; and variety concerns how multifarious are the formulaic sequences produced by each test-taker, which is the total types of formulaic sequences divided by the total number of formulaic sequences, and the “types” here refer to the total number of formulaic sequences excluding the repeated ones, rather than the so-called types based on structures or functions.

#### Measuring oral fluency

Since speed, breakdown, and repair fluency are closely related, there is not a clear boundary between indices measuring speed, breakdown, and repair fluency as they sometimes overlap. Nevertheless, for the sake of convenience and clarity, the present study has been sticking to the tripartite taxonomy, categorizing each index according to the dimension of fluency that is most reflected by it. In the present study, speed fluency was measured by speech rate (SR), articulation rate (AR), mean length of run (MLR), and phonation time ratio (PTR). Breakdown fluency was measured by three indices, namely frequency of silent pauses (FSP), mean length of silent pauses (MLP), and pause time ratio (PAR). Repair fluency was measured by frequency of all repairs per 60 s (FAR), frequency of false starts and reformulations per 60 s (FFR), frequency of partial or complete repetitions per 60 s (FRP), and frequency of self-corrections per 60 s (FSC).

Speed fluency and breakdown fluency were measured automatically with artificial intelligence techniques, while repair fluency was measured manually. To measure speed fluency and breakdown fluency, the recordings were analyzed with Praat, using a script developed by de Jong and Wempe (2009). Repair fluency was measured manually. The whole data set was coded with BFSU Qualitative Coder separately by three coders, according to Tree’s (1995) illustration of the indices of repair fluency. All the discrepancies were discussed until the agreement was reached. Then, indices of repairs were calculated with BFSU Qualitative Explorer.

#### Investigating the relationships

Quantitative analysis employed SPSS 25 for the exploration of the relationship between the use of formulaic sequences (frequency, proportion, and variety) and oral fluency (speed fluency, breakdown fluency, and repair fluency). Specifically, the present study employed not only Pearson correlation analysis, but also canonical correlational analysis (CCA), a statistical technique initially developed by Hotelling (1935, 1936) in the 1930s, which could investigate the relationship between two sets of variables, rather than two variables (Mandel et al., 2017). This technique is comprehensive and crucial, fitting many instances (Sherry and Henson, 2005). CCA could examine the relationship between two sets of variables by presenting the relationships between pairs of canonical variables, and these correlations are always positive, which should be the absolute value of their ordinary correlation coefficient (Basu and Mandal, 2010). Let *X* denote the first set of variables, which is *m* dimensional, and let *Y* denote the second set of variables, which is *n* dimensional. Let *m* ≤ *n*.Let Cov (*X*), Cov (*Y*) and Cov (*X, Y*) be denoted by $\sum_{11}^{m\times m},$ $\sum_{11}^{n\times n},$ and $\sum_{22}^{m\times n},$ respectively. Assume that the (m+n)×(m+n) dimensional square matrix


$$\sum =\left(\begin{array}{cc} \sum_{11} & \sum_{12}\\ \sum_{21} & \sum_{22} \end{array}\right)$$

is positive definite, where $\sum_{21}^{}=\sum_{12}^{T}.$ Canonical variables are the linear combinations of the variables in the whole variable set, namely:


U1=a1TX and V1=b1TY

in which $a_{1}$ and $b_{1}$ indicate *m* and *n* dimensional coefficient vectors. Then, the following equations could be derived:


$$\begin{array}{l} \mathrm{Var}\;\left(U_{1}\right)=a_{1}^{T}\sum_{11}^{}a_{1},\mathrm{Var}\;\left(V_{1}\right)=b_{1}^{T}\sum_{22}^{}b_{1},\\ \mathrm{cov}\;\left(U_{1,}\;V_{1}\right)=a_{1}^{T}\sum_{12}^{}b_{1} \end{array}$$

**Figure 2:**
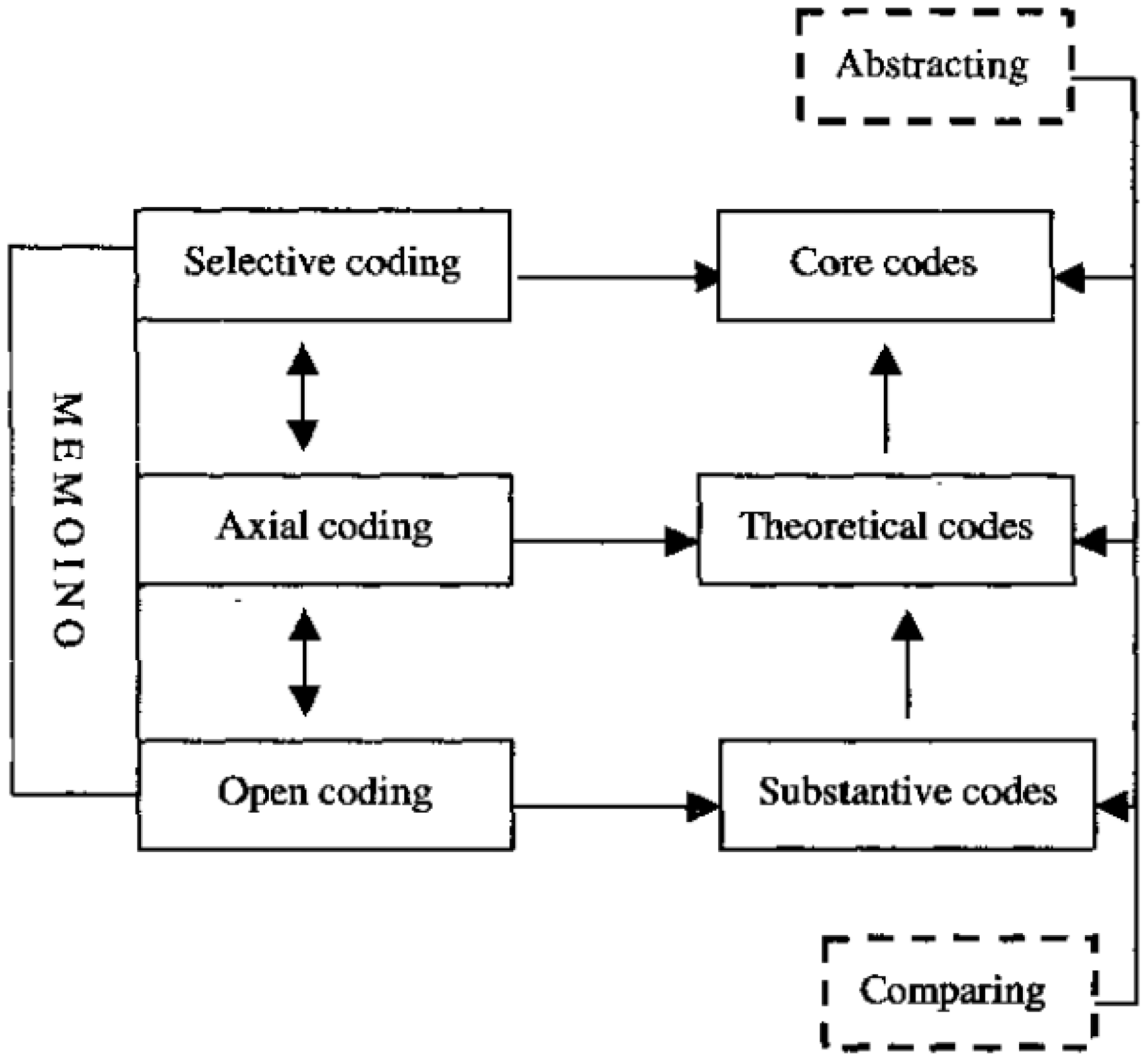
The coding procedure of qualitative analysis.

The equation of the canonical correlation is as follows:

$\mathrm{Corr}\;\left(U_{1,}\;V_{1}\right)=\frac{a_{1}^{T}\sum_{12}b_{1}}{\sqrt{a_{1}^{T}\sum_{11}a_{1}}\sqrt{b_{1}^{T}\sum_{22}b_{1}}}$ . Qualitative analysis followed the coding procedure in Grounded Theory Method (see Figure 2 for the procedure). The present study employed the software Dedoose,^3^ focusing on the roles of formulaic sequences in oral fluency. To be more exact, the present study investigated the speakers’ speed fluency, breakdown fluency, and repair fluency before and after uttering a formulaic sequence, which could reflect the relationship between the use of formulaic sequences and oral fluency. In the open coding, I marked every phenomenon related to the use of formulaic sequences or to oral fluency. I also investigated the commonalities between different codes, and considered about classifying them into different categories. In the axial coding, I reassembled the categories formed during the open coding. In the selective coding, the core category or the main theme of this qualitative research emerged, which should be “the mixed relationship between the use of formulaic sequences and oral fluency,” which was just what the present study intended to investigate. This core category was central to all the other categories identified in open coding and axial coding, occurring frequently in the data. The coding process was accompanied by memoing (memo keeping). I made notes for further explanation. I stopped data analysis when I had reached theoretical saturation and new data analysis did not elicit any new or significant insights. After the coding and memoing process, I went through the coded information, their different categories, and the generated themes again. These could provide rich information about the influence of formulaic sequences on oral fluency.

**Figure 3:**
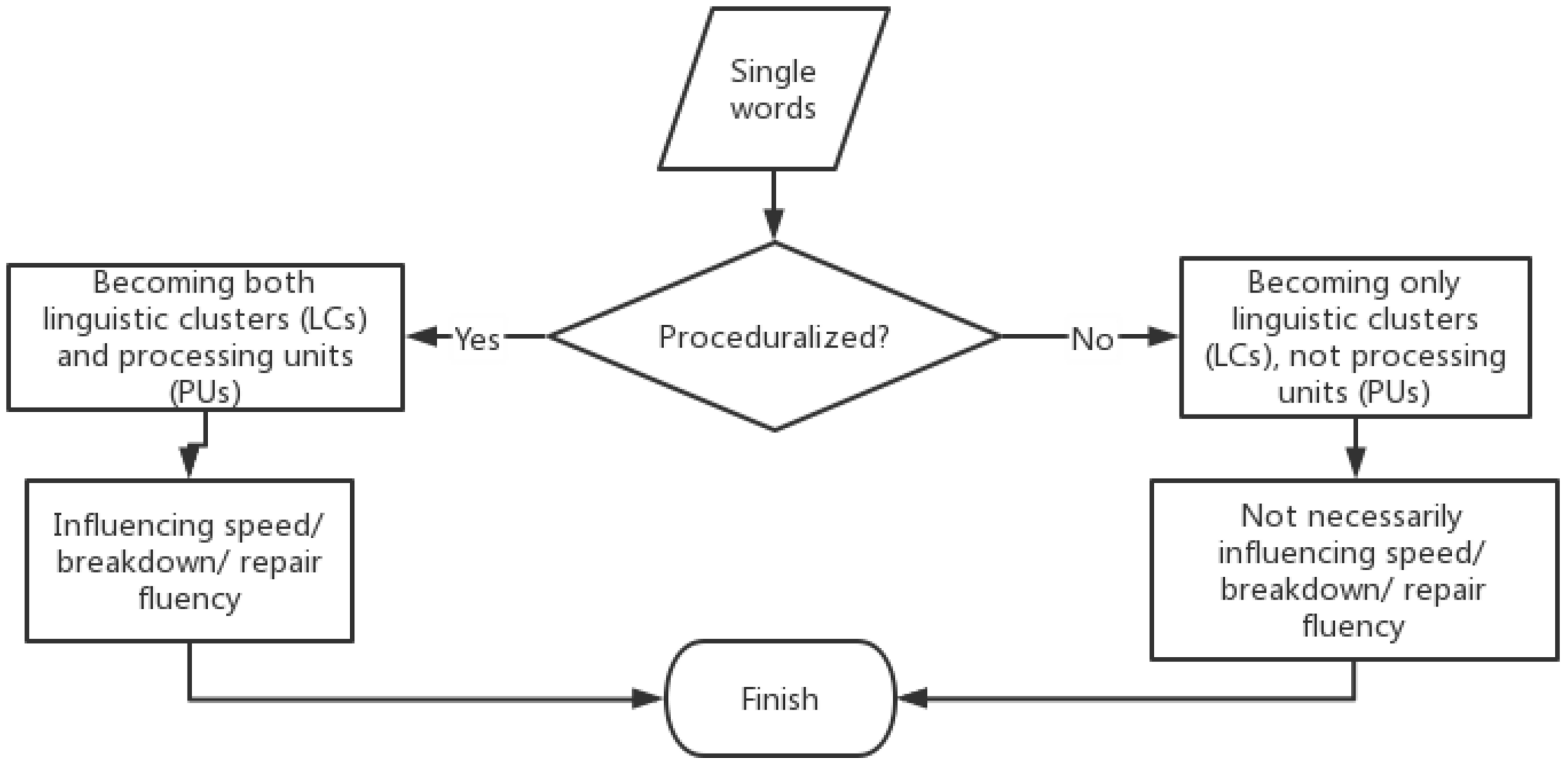
The framework of proceduralization of formulaic sequence use.

The general procedure of the whole framework is displayed as Figure 3. For the sake of validation regarding this data-driven approach, I first collected another sample containing 60 pieces of recordings from the corpus of SECCL (Spoken English Corpus of Chinese Learners), with similar features as the input in the present study, and tested the model in that sample. Then, I checked the fit of this model in the sample of the present study. The patterns were similar across these two samples.

## Results

Table 1 presents the descriptive results of the formulaic sequence variables as well as the oral fluency variables. Test-takers generally displayed a certain degree of distinction from each other in indices like the frequency of formulaic sequences, speech rate, articulation rate, and mean length of run. On the other hand, test-takers were relatively similar in indices like the proportion and variety of formulaic sequences, phonation time ratio, and pause time ratio.

**Table 1:** Descriptive statistics of the variables: FSs and oral fluency.

Variable	*N*	Minimum	Maximum	Mean	SD
Formulaic sequence variables					
Frequency	120	3.00	45.00	18.23	7.70
Proportion	120	0.01	0.12	0.06	0.02
Variety	120	0.52	1.00	0.80	0.12
Speed fluency variables					
SR	120	47.40	268.80	159.71	41.54
AR	120	64.80	279.00	209.22	40.91
PTR	120	0.39	1.00	0.77	0.15
MLR	120	3.11	673.00	42.43	100.64
Breakdown fluency variables					
FSP	120	0	41.12	16.75	10.16
PAR	120	0	0.61	0.23	0.15
MLP	120	0	5.08	0.89	0.57
Repair fluency variables					
FAR	120	0.33	18.57	4.57	3.40
FFR	120	0	4.40	1.05	0.92
FRP	120	0	13.93	2.41	2.55
FSC	120	0	3.98	1.10	0.85

SR, speech rate; AR, articulation rate; PTR, phonation time ratio; MLR, mean length of run; FSP, frequency of silent pauses; PAR, pause time ratio; MLP, mean length of silent pauses; FAR, frequency of all repairs; FFR, frequency of false starts and reformulations; FRP, frequency of repetitions; FSC, frequency of self-corrections.

### The relationship between formulaic sequence use and speed fluency

#### General correlations between indices of FSs and speed fluency

Table 2 presents the correlations between the three indices of formulaic sequences and the four indices of speed fluency. According to the statistics, significant positive correlations could only be found between frequency of FSs and speech rate (*r* = 0.559, *p* < 0.001), frequency of FSs and articulation rate (*r* = 0.391, *p* < 0.001), as well as frequency of FSs and phonation time ratio (*r* = 0.298, *p* < 0.01), respectively. Thus, total frequency of formulaic sequences is positively related to speed fluency to a large degree, total proportion of formulaic sequences only shows the tendency to be positively related to speed fluency, while total variety of formulaic sequences shows the tendency to be negatively related to speed fluency.

**Table 2:** Correlations between FSs and speed fluency.

Variable	Correlation	SR	AR	PTR	MLR
Frequency	*r*	**0.559**	**0.391**	**0.298**	0.161
	Sig.	**0.000**	**0.000**	**0.001**	0.079
Proportion	*r*	0.156	0.111	0.057	0.034
	Sig.	0.090	0.229	0.540	0.714
Variety	*r*	−0.100	−0.016	−0.115	−0.067
	Sig.	0.275	0.865	0.211	0.468

SR, speech rate; AR, articulation rate; PTR, phonation time ratio; MLR, mean length of run. Bold values indicate statistically significant correlations.

Table 3 summarizes the canonical correlation analysis of formulaic sequence use and speed fluency. As for the canonical correlation analysis of frequency of FSs and speed fluency variables’ set, this CCA (canonical correlation analysis) solution has a statistically significant correlation coefficient (RC) of 0.563 with an effect size of 31.7% (1–Wilk’s ƛ = 0.317) and the amount of 31.7% (RC2 = 0.5632 = 0. 317) shared variance. As for the canonical correlation analysis of proportion of FSs and speed fluency as well as of variety of FSs and speed fluency, no significance could be found.

**Table 3:** Summary of the canonical correlation analyses: FSs and speed fluency.

Canonical variable	Correlation	Eigen value	Wilks statistic	*F*	Num D.F.	Denom D.F.	Sig.
**Frequency**	**0.563**	**0.465**	**0.683**	**13.360**	**4.000**	**115.000**	**0.000**
Proportion	0.222	0.052	0.951	1.486	4.000	115.000	0.211
Variety	0.122	0.015	0.985	0.436	4.000	115.000	0.783

Bold values indicate statistically significant correlations.

To sum up, quantitative analysis indicates a significant positive relationship between frequency of formulaic sequences and speed fluency.

#### Case studies of roles of formulaic sequences in speed fluency

According to detailed analysis, the employment of formulaic sequences could promote speed fluency when retrieved and processed as a whole, but failed to promote speed fluency when retrieved and processed in a word-by-word manner, losing their processing advantages.

As has been mentioned, there are actually two approaches to formulaic sequences, namely speaker-external and speaker-internal (Wray, 2008). Based on this distinction, Myles and Cordier (2017) drew a distinction between linguistic clusters (LCs), which are speaker-external formulaic sequences, and processing units (PUs), which are speaker-internal formulaic sequences.

As the present study mainly focuses on the speaker-external approach, all the formulaic sequences in the present study are linguistic clusters (LC), but they did not always function as processing units (PU). When a formulaic sequence functioned both as a linguistic cluster (LC) and a processing unit (PU) in the present study, it was stored and retrieved holistically, showcasing proceduralization and processing advantages, promoting speed and breakdown fluency. On the contrary, when a formulaic sequence functioned only as a linguistic cluster (LC) but not as a processing unit (PU), then it was retrieved and processed in a word-by-word manner, similar to that of the non-formulaic language, losing processing advantages, and did not promote speed and breakdown fluency.

##### Facilitative effects of FSs (PUs) on speed fluency

Formulaic sequences were often uttered at a faster speed. Speakers often uttered formulaic sequences faster than other words. Consider the following example:

[B2-3] But there… and there were not any snack in my house when I *got around* it.

In example [B2-3], the speaker B2 maintained a slow speed when uttering most of the words in the sentence “and there were not any snack in my house,” but when she began to utter the formulaic sequence “*got around*,” her speed rose immediately.

It should also be noted that the facilitative effects of formulaic sequences were often not influenced by their positions in a sentence. No matter a formulaic sequence occurred at the beginning or end of a sentence, it could promote speed, as long as it was retrieved and processed as a whole. The processing advantages of formulaic sequences would not disappear along with the change of their positions. Consider the following group of examples:

[C3-1] Everything was fine *at that moment*.

[C3-4] *At that moment*, I felt a sense of relief.

In examples [C3-1] and [C3-4], the speaker C3 employed the formulaic sequence “*at that moment*” twice in total in his speech, one of which at the beginning of a sentence, and the other at the end of a sentence. In both cases, the formulaic sequence “*at that moment*” was uttered much faster than other sentences, demonstrating the facilitative effect of formulaic sequences on speed, regardless of their location in a sentence.

The facilitative effects of formulaic sequences on speed fluency were sometimes due to their easiness of pronunciation in the articulation stage. The speakers sometimes obscured the pronunciation of a component of the word, thereby pronouncing them faster, consistent with previous findings (Kormos, 2006; Skehan, 2014). Consider the following example:

[B3-5] But after that, after we have our model planes *at home*, I felt… I started to feel guilty about it.

In example [B3-5], when uttering the formulaic sequence “*at home*,” the speaker B3 obscured the sound of [t], which made the utterance of “*at home*” even faster, greatly increasing speed, contributing to speed fluency.

Speakers sometimes chose to link a few formulaic sequences so as to extend their length of run, creating a longer run, consistent with the findings of Wood (2010). In this way, the use of formulaic sequences could contribute to the increase of the mean length of run, thereby contributing to speed fluency. Consider the following example:

[B6-3] And he *picked me up as usual*…

In example [B6-3], the speaker B6 strung together two formulaic sequences, namely “*picked me up*” and “*as usual*,” hence creating a longer run, namely “*picked me up as usual*,” which contained no pauses, posing a contrast to the utterance of “and he,” as he paused for 1 s, respectively, before and after uttering “he.”

The string of two or more formulaic sequences could benefit speakers who often made pausing or repairs in their speech. This would be further illustrated in the next section when discussing the influence of formulaic sequences on breakdown fluency. Consider the following example:

[C8-1] And after that, *I know that* a hardworking student do not *have to go to the library every day*, but *have to* be hardworking…

In example [C8-1], the speaker used five formulaic sequences in this entry, three of which were strung together to make a longer run, namely “*have to go to the library every day*.” In her speech, this speaker C8 frequently paused and made repairs. But thanks to these formulaic sequences in this entry, she made no pausing or repairs and uttered this sentence faster.

To sum up, the use of formulaic sequences could often facilitate speed fluency by increasing rate and amount of speech. Formulaic sequences were often uttered at a faster rate or strung up together to create a longer run. The facilitative effects were not influenced by the position of formulaic sequences in a sentence. Formulaic sequences could promote speed fluency for both fast speakers and slow speakers, but the facilitative effects were especially prominent for slow speakers. The facilitative effects were sometimes due to the easiness of pronunciation.

##### Loss of facilitative effects of FSs (LCs) on speed fluency

Despite the processing advantages of formulaic sequences reported above, it is also possible for formulaic sequences to lose their processing advantages, when they functioned as only linguistic clusters (LCs), failing to promote speed. Actually, in the present study, those speakers who performed not so well in speed fluency, or in other words, whose indices of speed fluency were among the last 50% of all speakers, were more likely to retrieve and process formulaic sequences in a word-by-word manner. Consider the following example:

[D1-3] And from the things I get a good lesson we should *care for*… for everything.

In example [D1-3], the speaker D1, whose indices of speed fluency were among the last 20% of all speakers, obviously retrieved the formulaic sequence “*care for*” in a word-by-word manner, rather than holistically. She repeated the word “for” as a filler, instead of repeating the formulaic sequence “*care for*” as a filler. This suggests that she did not regard “*care for*” as a prefabricated chunk, or as a whole. Consequently, the utterance of “*care for*” was slow, even more slowly than other words in this sentence, showing no processing advantage to facilitate speed.

In short, formulaic sequences failed to facilitate speed fluency when they were retrieved and processed in a word-by-word manner. This phenomenon usually occurred in the performance of those slow speakers.

### The relationship between formulaic sequence use and breakdown fluency

#### General correlations between indices of FSs and breakdown fluency

Table 4 presents the correlations between the three indices of formulaic sequences and the three indices of breakdown fluency. According to the statistics, significant negative correlations could only be found between frequency of FSs and pause time ratio (*r* = −0.298, *p* < 0.01). Thus, total frequency of formulaic sequences is negatively related to pausing to a certain degree, while proportion of FSs and variety of FSs show a mixed pattern regarding their relationships with pausing.

**Table 4:** Correlations between FSs and breakdown fluency.

Variable	Correlation	FSP	PAR	MLP
Frequency	*r*	−0.148	**−0.298**	−0.123
	Sig.	0.107	**0.001**	0.181
Proportion	*r*	−0.033	−0.057	0.027
	Sig.	0.723	0.540	0.767
Variety	*r*	0.084	0.115	−0.045
	Sig.	0.364	0.211	0.628

FS, frequency of silent pauses; PAR, pause time ratio; MLP, mean length of silent pauses. Bold values indicate statistically significant correlations.

Table 5 summarizes the canonical correlation analysis of formulaic sequence use and breakdown fluency. As for the canonical correlation analysis of frequency of FSs and breakdown fluency variables’ set, this CCA (canonical correlation analysis) solution has a statistically significant correlation coefficient (RC) of 0.360 with an effect size of 13% (1–Wilk’s ƛ = 0.130) and the amount of 13% (RC2 = 0.3602 = 0.130) shared variance. As for the canonical correlation analysis of proportion of FSs and breakdown fluency as well as of variety of FSs and breakdown fluency, no significance could be found.

**Table 5:** Summary of the canonical correlation analyses: FSs and breakdown fluency.

Canonical variable	Correlation	Eigenvalue	Wilks statistic	*F*	Num D.F.	Denom D.F.	Sig.
**Frequency**	**0.360**	**0.149**	**0.870**	**5.753**	**3.000**	**116.000**	**0.001**
Proportion	0.082	0.007	0.993	0.259	3.000	116.000	0.855
Variety	0.139	0.020	0.981	0.766	3.000	116.000	0.515

Bold values indicate statistically significant correlations.

To sum up, quantitative analysis indicates a significant negative relationship between frequency of formulaic sequences and pausing.

#### Case studies of roles of formulaic sequences in breakdown fluency

According to detailed analysis, the employment of formulaic sequences could help reduce pausing when retrieved and processed as a whole, but failed to reduce pausing when retrieved and processed in a word-by-word manner. On the former condition, the formulaic sequences functioned as both linguistic clusters (LCs) and processing units (PUs), while on the latter condition, the formulaic sequences only functioned as linguistic clusters (LCs).

##### Facilitative effects of FSs (PUs) on breakdown fluency

The employment of these formulaic sequences could reduce pausing, reflecting their negative relationship with pausing. The effects of formulaic sequences on reduction of pausing are reflected in two aspects. For one thing, formulaic sequences could help reduce the number of pauses. For another, formulaic sequences could help shorten the length of pausing. Consider the following two examples:

[B4-2] …that was my younger sister’s mistake. After *getting home* that night, I was very guilty.

In example [B4-2], the speaker B4 paused a few times when uttering “that was my younger sister’s mistake” as well as after uttering “after.” However, when he retrieved “*getting home*,” he uttered it smoothly without any pauses.

[C10-1] And unfortunately, I took her offer as (2 s) … *as usual*.

In example [C10-1], the speaker C10 might originally intend to say something with the meaning of “take something for granted.” However, he failed to come up with a phrase like that, leading to a pause of 2 s after the utterance of “took her offer as.” Then, he had to find other expressions to shorten the length of pause, so he finally employed the formulaic sequence “*as usual*,” which was uttered with a fast speed and no pausing in between.

The two examples above showcased the effects of formulaic sequences on the reduction of the number of pauses (Example [B4-2]) and on the shortening of the length of pausing (Example [C10-1]) respectively.

Besides, after the utterance of formulaic sequences, speakers could sometimes uttered the later words smoothly, with few pauses. Consider the following example:

[B5-3] I made so many (2 s) mistakes *in my life* that it is uncountable.

In example [B5-3], the speaker paused frequently before uttering the formulaic sequence “in my life.” He even paused for 2 s between the utterances of “many” and “mistakes.” When uttering the formulaic sequence “*in my life*,” however, the speech became quite smooth, displaying no pausing. Also, after uttering “*in my life*,” the speaker also uttered “that it is uncountable” much more smoothly than “I made so many mistakes,” which might be due to the processing time saved from the holistic retrieval and processing of “*in my life*.”

To sum up, formulaic sequences could help facilitate breakdown fluency by reducing the number and length of pauses. The use of formulaic sequences helped reduce pausing not only during their utterance, but also after their utterance. Speakers sometimes borrowed formulaic sequences from task prompts, and these formulaic sequences were usually uttered with no pausing, facilitating breakdown fluency.

##### Loss of facilitative effects of FSs (LCs) on breakdown fluency

As has been mentioned in Section “Loss of Facilitative Effects of FSs (LCs) on Speed Fluency”, it is possible for formulaic sequences to lose their processing advantages when they functioned as only linguistic clusters (LCs), failing to reduce pausing. Again, in the present study, those speakers who performed not so well in breakdown fluency, or in other words, whose indices of breakdown fluency were among the last 50% of all speakers, were more likely to retrieve and process formulaic sequences in a word-by-word manner. It should be noted that the indices of breakdown fluency of all speakers were sequenced from the smallest numbers to the largest numbers. Thus, the so-called “last 50%” means that the numbers themselves were larger than the “first 50%,” indicating that these speakers were among the last 50% regarding their breakdown fluency performance. Consider the following example:

[C22-2] I *took (1 s) part in* a… in a speaking competition.

In example [C22-2], the speaker C22, whose indices of breakdown fluency were among the last 20% of all speakers, retrieved the formulaic sequence “*took part in*” in a word-by-word manner, since she paused for 1 s between the utterance of “took” and that of “part,” suggesting that she actually retrieved “took” and “park” separately, rather than as a whole.

In short, formulaic sequences failed to facilitate breakdown fluency when they were retrieved and processed in a word-by-word manner. This phenomenon usually occurred in the performance of those speakers with more pausing.

### The relationship between formulaic sequence use and repair fluency

#### General correlations between indices of FSs and repair fluency

Table 6 presents the correlations between the three indices of formulaic sequences and the three indices of repair fluency. According to the statistics, significant negative correlations could only be found between variety of FSs and frequency of all repairs (*r* = −0.285, *p* < 0.01), variety of FSs and frequency of false starts and reformulations (*r* = −0.227, *p* < 0.05), as well as variety of FSs and frequency of repetitions (*r* = −0.255, *p* < 0.01). Thus, total variety of formulaic sequences is negatively related to repairing to a certain degree.

**Table 6:** Correlations between FSs and repair fluency.

Variable	Correlation	FAR	FFR	FRP	FSC
Frequency	*r*	−0.035	0.152	−0.104	0.007
	Sig.	0.705	0.098	0.260	0.939
Proportion	*r*	−0.108	0.040	−0.138	−0.061
	Sig.	0.240	0.667	0.132	0.509
Variety	*r*	**−0.285**	**−0.227**	**−0.255**	−0.129
	Sig.	**0.002**	**0.013**	**0.005**	0.160

FAR, frequency of all repairs; FFR, frequency of false starts and reformulations; FRP, frequency of repetitions; FSC, frequency of self-corrections. Bold values indicate statistically significant correlations.

Table 7 summarizes the canonical correlation analysis of formulaic sequence use and repair fluency. As for the canonical correlation analysis of variety of FSs and repair fluency variables’ set, this CCA (canonical correlation analysis) solution has a statistically significant correlation coefficient (RC) of 0.292 with an effect size of 8.5% (1- Wilk’s ƛ = 0.085) and the amount of 8.5% (RC2 = 0.2922 = 0.085) shared variance. As for the canonical correlation analysis of frequency of FSs and repair fluency as well as of proportion of FSs and repair fluency, no significance could be found.

**Table 7:** Summary of the canonical correlation analyses: FSs and repair fluency.

Canonical variable	Correlation	Eigen value	Wilks statistic	*F*	Num D.F.	Denom D.F.	Sig.
Frequency	0.237	0.060	0.944	1.718	4.000	115.000	0.151
Proportion	0.176	0.032	0.969	0.920	4.000	115.000	0.455
**Variety**	**0.292**	**0.093**	**0.915**	**2.678**	**4.000**	**115.000**	**0.035**

Bold values indicate statistically significant correlations.

To sum up, quantitative analysis indicates a significant negative relationship between variety of formulaic sequences and repairing.

#### Case studies of roles of formulaic sequences in repair fluency

Similar to the cases of speed and breakdown fluency, for repair fluency, formulaic sequences also had facilitative effects on the reduction of repairing to a certain degree. However, what distinguished the case of repair fluency from those of speed and breakdown fluency is that formulaic sequences were often entwined with repair phenomena, washing away their facilitative effects on the reduction of repairing. According to detailed analysis, participants often made correction and reformulation of formulaic sequences, repeated the same formulaic sequence as a filler, and reused the same formulaic sequence in self-correction or reformulation. All these led to the decrease of the variety of formulaic sequences along with the increase of repairs, explaining the negative relationship between the variety of formulaic sequences and repairing.

##### Facilitative effects of FSs on repair fluency

The employment of formulaic sequences sometimes helped to reduce the number of repairs, reflecting the facilitative effects on repair fluency. Consider the following example:

[B20-3] Because I did not have any *pocket money at that time*.

In example [B20-3], the speaker strung together two formulaic sequences, namely “*pocket money*” and “*at that time*” so as to create a longer run of “*pocket money at that time*.” This speaker B20 often made repairs in her speech. However, the utterance of this run constituting “*pocket money*” and “*at that time*” was very smooth, with no repairs.

In this way, the use of formulaic sequences did help to reduce repairing from time to time, promoting repair fluency.

##### Loss of facilitative effects of the reused FSs on repair fluency

When making self-corrections, speakers sometimes did not restart with the targeted information alone, but reused the same formulaic sequences, uttering them again. In other words, they sometimes repeated formulaic sequences together with the targeted information. Consider the following example:

[D18-1] I should not *quarrel with* her… *quarrel with* him.

In example [D18-1], the speaker D18 mistakenly uttered “her” instead of “him,” after uttering the formulaic sequence “*quarrel with*.” When she made the self-correction, she did not directly utter “(*quarrel with* her…) him” but uttered “(*quarrel with* her…) *quarrel with* him,” reusing the formulaic sequence “*quarrel with*.”

Similarly, when speakers decided to abort their original expressions and restarted their utterances, they sometimes did not restart with the targeted information alone, but reused the same formulaic sequences in their reformulations. Consider the following example:

[B15-1] The elevator *have to* for… *have to* stop on each floor…

In example [B15-1], the speaker B15 originally intended to say something beginning with “*have to* for,” but changed his mind and decided to switch to another expression, namely “stop on each floor.” However, he did not directly utter “(*have to* for…) stop on each floor,” but reused the formulaic sequence “*have to*,” and uttered “(*have to* for…) have to stop on each floor.”

To sum up, speakers often employed formulaic sequences when making self-corrections, restarting utterances, or switching to new information. During these processes, the use of formulaic sequences generally did not have extra facilitative effects on repair fluency, since these formulaic sequences were already being used in self-corrections or reformulations.

##### Negative effects of the filler FSs on repair fluency

When speakers failed to come up with words, they sometimes repeated formulaic sequences or those expressions containing formulaic sequences. On these occasions, the formulaic sequences or those expressions containing formulaic sequences actually acted as “filler words.” This process might help avoid a pause, thereby mirroring the facilitative effects of formulaic sequences on breakdown fluency. However, the frequency of repetitions was counted as an index of repairing, so that the use (repetition) of formulaic sequences here actually had negative effects on repair fluency.

The repetition of the same formulaic sequences could sometimes help avoid a pause, which could also help illustrate the facilitative effect of formulaic sequences on reducing the frequency of pauses. Consider the following example:

[B6-2] And I *went out*… *went out* from school.

In example [B6-2], the speaker B6 did not know what to say after uttering the formulaic sequence “*went out*,” so he repeated the formulaic sequence “*went out*” to buy time, avoiding a pause here.

On some other occasions, the repetition of the same formulaic sequences could not avoid pausing, but could help shorten the length of pausing, which could illustrate the facilitative effect of formulaic sequences on shortening the length of pausing. Consider the following example:

[C15-2] I have earned about 100 money and I happily *went to* a supermarket to *look for*… *look for* the best car and I bought it.

In example [C15-2], the speaker C15 uttered the formulaic sequence “*went to*” with a fast speed and no pausing. But after he uttered the formulaic sequence “*look for*,” he failed to come up with what he intended to say and paused. Consequently, he repeated the formulaic sequence “*look for*” as a filler, so as to buy time for later processing shortening the length of the pause here.

In short, speakers in the present study sometimes repeated formulaic sequences. This did lead to the reduction of pausing, but this also led to the increase of repairing.

##### Negative effects of the corrected FSs on repair fluency

Speakers sometimes made mistakes when using formulaic sequences, and then they had to correct or reformulate their expressions. The frequency of false starts and reformulations as well as the frequency of self-corrections were counted as indices of repairing, so that the use of formulaic sequences here actually had negative effects on repair fluency. Consider the following example:

[B16-2] So in the first month of the test, my *report card*… *report card* was very bad.

In example [B16-2], when the speaker B16 uttered “*report card*” for the first time, he pronounced the word “report” incorrectly, with the wrong stress location. Then he made a self-correction and uttered “*report card*” again, with a correct pronunciation this time. In this way, the speaker actually used the same formulaic sequence “*report card*” twice, reducing variety of formulaic sequences while increasing repairs.

To sum up, the use of formulaic sequences could bring about self-corrections and reformulations when they contained mistakes. Under these circumstances, they actually had negative effects on repair fluency.

## Discussion

Quantitative analysis indicates significant relationships between formulaic sequence use and oral fluency. For speed fluency, quantitative analysis indicates a significant positive relationship between frequency of formulaic sequences and speed fluency, proving the findings of many of the previous studies (Wood, 2009, 2010; McGuire and Larson-Hall, 2017; Thomson, 2017; Carrol and Conklin, 2020; Saito, 2020; Tavakoli and Uchihara, 2020; Ogawa, 2021; Xuan et al., 2021). For breakdown fluency, quantitative analysis indicates a significant negative relationship between frequency of formulaic sequences and pausing, proving the findings of many of the previous studies (Tavakoli, 2011; Carrol and Conklin, 2020; Saito, 2020; Tavakoli and Uchihara, 2020; Yan, 2020). For repair fluency, quantitative analysis indicates a significant negative relationship between variety of formulaic sequences and repairing, which was hardly explored in previous research.

Qualitative analysis suggests the roles and functions of formulaic sequences through proceduralization. The use of formulaic sequences promoted speed fluency and reduced pausing when retrieved and processed holistically, but lost their processing advantages when retrieved and processed in a word-by-word manner. Some formulaic sequences seemed to be retrieved and processed as wholes (Pawley and Syder, 1983; Peters, 1983; Weinert, 1995; Wray, 2002), in line with the psycholinguistic research evidence indicating that formulaic language is usually stored and retrieved as individual units, allowing for the quick and convenient processing of information (Ellis, 2002; Siyanova-Chanturia and Van Lancker Sidtis, 2018). The use of formulaic sequences was actually proceduralized (Anderson, 1983). Then, unnecessary and cognitively expensive syntactic operations could be bypassed (Pawley and Syder, 1983; Nattinger and DeCarrico, 1992; Wray, 2002). As a consequence, formulaic sequences were processed faster than separate words (Jiang and Nekrasova, 2007; Conklin and Schmitt, 2008; Siyanova-Chanturia et al., 2011; Tremblay et al., 2011). In this way, the employment of these formulaic sequences could promote speed, facilitating speed fluency, through the proceduralization.

Moreover, thanks to the proceduralization, the holistic retrieval and processing of formulaic sequences could help free up the attentional resources for other aspects of language production, reducing the amount of language planning and processing, thereby facilitating oral production (Kormos, 2006; Skehan, 2009, 2014; Siyanova-Chanturia and Van Lancker Sidtis, 2018; Tavakoli and Uchihara, 2020). Besides, since the retrieval of formulaic sequences helped save processing time for the preparation of other processing needs (Skehan, 1998; Boers et al., 2006), speakers could have enough time for the retrieval and processing of the words after the utterance of formulaic sequences. Consequently, they could sometimes utter the later words smoothly, with few pauses. In this way, the employment of these formulaic sequences could reduce pausing, reflecting their negative relationship with pausing.

The facilitative effects of formulaic sequences on speed or breakdown fluency are shown in different stages of speech production, reflecting proceduralization. As has been mentioned, among the three stages in language production, namely conceptualization, formulation, and articulation (Levelt, 1989; Kormos, 2006), formulaic sequences could facilitate speed fluency in formulation and articulation stages, when they were treated as a whole. At the formulation stage, speakers retrieved formulaic sequences holistically. This could be reflected in speakers’ faster utterance of formulaic sequences in the present study, because speakers did not have to slow down to retrieve the single words constituting those formulaic sequences. Neither did they have to pause to think about what they should retrieve when uttering a formulaic sequence. Sometimes, the processing load for a formulaic sequence could be similar to that for a single word. Component words of these formulaic sequences were retrieved and processed autonomously, showcasing proceduralization (Anderson, 1983). When speakers strung together several formulaic sequences, the effects of proceduralization could be strengthened, and the retrieval of that string could save even more time and effort, further contributing to speed fluency. Also, holistic retrieval could help reduce the processing load, so that speakers are able to use remaining attentional resources for other aspects of language processing, such as syntactic and phonological encoding (Kormos, 2006), which also helps improve speed fluency. Then, at articulation stage, formulaic sequences were often pronounced in an autonomous way, and left no space for pausing, since they could involve reduction of phonetic durations (e.g., deletion of [t] in “I do not know”; Bybee and Scheibman, 1999) when they were processed holistically, rather than articulating every constituent word in full form (e.g., pronouncing the sound of [t] in “I do not know”) in word-by-word processing.

Despite the processing advantages of formulaic sequences reported above, it should be noted that the proceduralization could not be the same for everyone. As a matter of fact, a word sequence processed holistically by one person may not be processed holistically by another, and the matter of holistic processing may be a matter of degree (Boers et al., 2006). It is possible for nonnative speakers to retrieve or process formulaic sequences in a word-by-word manner, rather than as a whole (Conklin and Schmitt, 2012), which occurred in speakers’ performance from time to time in the present study. On these conditions, formulaic sequences often lost their processing advantages, failing to promote speed.

Speakers sometimes did not resort to holistic processing, but to analytical processing when retrieving formulaic sequences (Wray, 1992, 2002), failing to complete proceduralization. Instead of retrieving prefabricated formulaic sequences as a whole, they actually broke down the whole string into words, and retrieved them separately based on grammatical rules. They were usually at the cognitive or associate stages, failing to reach the autonomous stage of proceduralization (Anderson, 1983). They had to devote much attention to the analysis of these constituent words. Consequently, the word-by-word retrieval and processing of formulaic sequences occupied much attention and added to the cognitive load.

These findings could explain why the overall proportion of formulaic sequences was not significantly related to speed or breakdown fluency, while the overall frequency of formulaic sequences was significantly positively related to speed fluency and negatively related to pausing. Those speakers who performed well in speed and breakdown fluency were more likely to resort to the idiom principle (Sinclair, 1991) when using formulaic sequences, which more often functioned as both linguistic clusters (LC) and processing units (PU), retrieved and processed holistically. Thus, these fluent speakers could produce more words, including more formulaic sequences, which could help explain the positive relationship between the overall frequency of formulaic sequences and speed fluency as well as the negative relationship between the overall frequency of formulaic sequences and pausing, reaching a significant level. By contrast, those speakers who performed not so well in speed and breakdown fluency were more likely to resort to the open-choice principle (Sinclair, 1991) when using formulaic sequences, which more often functioned as only linguistic clusters (LC), retrieved and processed in a word-by-word manner. Although the proportion of formulaic sequences they produced was not significantly less than those produced by fluent speakers, these formulaic sequences did not always showcase processing advantages. As a consequence, these less fluent speakers did not produce so many words, so that the total number of formulaic sequences produced by them were significantly fewer than those produced by more fluent speakers.

Simply put, as for speed and breakdown fluency, the key problem in the employment of formulaic sequences of those less fluent speakers does not lie in the quantity of formulaic sequences, but lies in the quality of formulaic sequences. It is not that those less fluent speakers were not so prone to use formulaic sequences as fluent speakers, but that they failed to use formulaic sequences properly to give full play to the processing advantages of formulaic sequences.

The negative relationship between variety of formulaic sequences and repairing does not mean that more varied usage of formulaic sequences could help reduce repairing. Instead, this negative relationship resulted from speakers’ reuse of the same formulaic sequence in self-correction or reformulation, repetition of the same formulaic sequence as a filler, and correction and reformulation of formulaic sequences. All these could lead to the decrease of variety of formulaic sequences and increase of repairs at the same time, explaining the negative relationship between the variety of formulaic sequences and repairing.

The proceduralization of formulaic sequence use did facilitate repair fluency on some occasions, but on other conditions, such as those mentioned in the previous paragraph, the use of formulaic sequences lost their facilitative effects or even had negative effects on repair fluency. In this way, the original facilitative effects on repair fluency was washed away, explaining the absence of significant relationship between formulaic sequence use and repair fluency.

First, when making self-corrections and reformulations, speakers repeated the proceduralized information (i.e., formulaic sequences), which could help them compensate for the limited capacity of working memory (Anderson, 1983), freeing up processing load, and buying more processing time for the preparation of their corrected or restarted information (Skehan, 1998; Boers et al., 2006). However, these facilitative effects could only be reflected in speed or breakdown fluency, rather than in repair fluency, since this procedure was already embedded in repairing.

Second, although the holistic retrieval of some formulaic sequences did help free up processing load and contribute to language production through proceduralization, they themselves could also contain mistakes and lead to self-corrections, exerting negative impact on repair fluency.

Third, speakers sometimes repeated the proceduralized information (i.e., formulaic sequences) to buy more processing time for the preparation of other processing needs (Skehan, 1998; Boers et al., 2006). The nature of formulaic sequence retrieval allows a single short formulaic sequence to be uttered many times in one sentence relatively effortlessly, so as to ensure the production of a large string of discourse with minimal strain on memory capacity (Wood, 2010). When speakers repeated formulaic sequences as “filler words,” they did not have to do information processing. This could reduce the load on the attentional resources, freeing up the attentional resources for the retrieval of new information after the utterance of formulaic sequences (Kormos, 2006; Skehan, 2009, 2014; Siyanova-Chanturia and Van Lancker Sidtis, 2018; Tavakoli and Uchihara, 2020). In this way, speakers could have adequate time to think about what they should say without slowing down or pausing. However, the improvement of speed or breakdown fluency was not achieved without price. By doing so, speakers actually sacrificed their repair fluency for speed or breakdown fluency to some extent, since the repetition of formulaic sequences led to the increase of repetitions in their speech, one of the indices to measure repairing. As a result, their repair fluency was negative affected.

**Figure 4:**
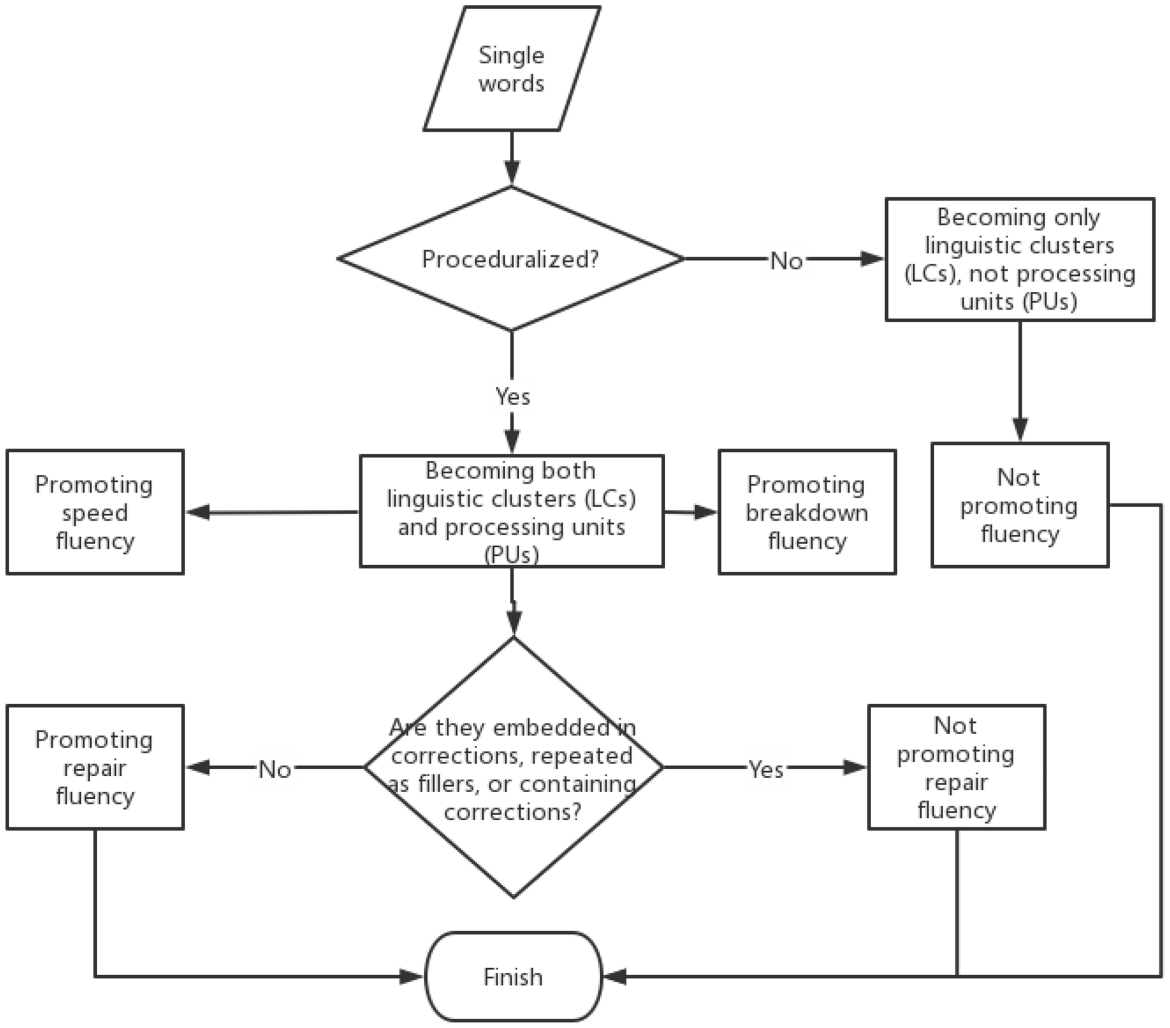
A new framework of proceduralization of formulaic sequence use.

Based on the results of quantitative and qualitative analyses, a new framework of proceduralization of formulaic sequence use could be constructed (see Figure 4 for the new framework). This framework can be employed, tested, or modified in future studies.

## Conclusion

The present study investigated the relationship between Chinese EFL learners’ use of formulaic sequences and oral fluency, reflecting the roles of psycholinguistic theories related to proceduralization in language learning. Total frequency of formulaic sequences was positively related to speed and negatively related to pausing, while total variety of formulaic sequences was negatively related to repairing, and all these relationships reached significant levels. Formulaic sequences could promote speed and reduce pausing when retrieved holistically, but sometimes lose their processing advantages when retrieved and processed in a word-by-word manner. Less fluent speakers are much more likely to retrieve and process formulaic sequences in a word-by-word manner, and their key problem lies in the quality rather than quantity of formulaic sequences. Formulaic sequences are also reused in self-corrections, repeated as fillers, corrected or reformulated, reducing variety while increasing repairs. These also neutralize the facilitative effects of formulaic sequences on repair fluency and could mirror speakers’ occasional tendency to sacrifice repair fluency for the improvement of speed and breakdown fluency when using formulaic sequences. Theoretically, the present study reflects the roles of psycholinguistic theories of proceduralization in teaching and learning by unpacking the relationship between formulaic sequence use and three dimensions of oral fluency. A new framework of the proceduralization of formulaic sequence use was also constructed accordingly (see Figure 4 for details). Pedagogically, the present study suggests that teachers could help students familiarize themselves with formulaic sequences. The present study has found that for those less fluent speakers, the key problem in the use of formulaic sequences does not lie in quantity, but in quality. They do use many formulaic sequences, but in their speech, formulaic sequences are sometimes retrieved in a word-by-word manner.

Despite the advantages and contributions mentioned above, the main disadvantage or limitation of the study is that it does not consider the categories of formulaic sequences or the oral proficiency of speakers when exploring the relationship between formulaic sequence use and oral fluency. Future research could consider these factors that might interplay with the functions of formulaic sequences in the mechanism of proceduralization, helping students know how to further improve their oral fluency, which is closely related to their mental state or mental health.

## Data availability statement

The raw data supporting the conclusions of this article will be made available by the authors, without undue reservation.

## Ethics statement

Ethical review and approval was not required for the study on human participants in accordance with the local legislation and institutional requirements. Written informed consent for participation was not required for this study in accordance with the national legislation and the institutional requirements.

## Author contributions

The author confirms being the sole contributor of this work and has approved it for publication.

## Conflict of interest

The author declares that the research was conducted in the absence of any commercial or financial relationships that could be construed as a potential conflict of interest.

## Publisher’s note

All claims expressed in this article are solely those of the authors and do not necessarily represent those of their affiliated organizations, or those of the publisher, the editors and the reviewers. Any product that may be evaluated in this article, or claim that may be made by its manufacturer, is not guaranteed or endorsed by the publisher.

## Footnotes

^1^ http://otter.ai

^2^ http://www.ldoceonline.com

^3^ www.dedoose.com/
